# Hyperbaric oxygen treatment is associated with a decrease in cytokine levels in patients with necrotizing soft‐tissue infection

**DOI:** 10.14814/phy2.14757

**Published:** 2021-03-14

**Authors:** Morten Hedetoft, Peter Garred, Martin Bruun Madsen, Ole Hyldegaard

**Affiliations:** ^1^ Department of Anaesthesia Hyperbaric Unit Rigshospitalet Copenhagen University Hospital Copenhagen Denmark; ^2^ Laboratory of Molecular Medicine Department of Clinical Immunology Section 7631 Rigshospitalet Copenhagen University Hospital Copenhagen Denmark; ^3^ Department of Intensive Care Rigshospitalet Copenhagen University Hospital Copenhagen Denmark

**Keywords:** cytokine, group A‐Streptococcus, anaerobic infection, hyperbaric oxygen treatment, necrotizing soft‐tissue infection, outcome, survival

## Abstract

**Background:**

The pathophysiological understanding of the inflammatory response in necrotizing soft‐tissue infection (NSTI) and its impact on clinical progression and outcomes are not resolved. Hyperbaric oxygen (HBO_2_) treatment serves as an adjunctive treatment; however, its immunomodulatory effects in the treatment of NSTI remains unknown. Accordingly, we evaluated fluctuations in inflammatory markers during courses of HBO_2_ treatment and assessed the overall inflammatory response during the first 3 days after admission.

**Methods:**

In 242 patients with NSTI, we measured plasma TNF‐α, IL‐1β, IL‐6, IL‐10, and granulocyte colony‐stimulating factor (G‐CSF) upon admission and daily for three days, and before/after HBO^2^ in the 209 patients recieving HBO^2^. We assessed the severity of disease by Simplified Acute Physiology Score (SAPS) II, SOFA score, and blood lactate.

**Results:**

In paired analyses, HBO_2_ treatment was associated with a decrease in IL‐6 in patients with *Group A*‐*Streptococcus* NSTI (first HBO_2_ treatment, median difference −29.5 pg/ml; second HBO_2_ treatment, median difference −7.6 pg/ml), and overall a decrease in G‐CSF (first HBO_2_ treatment, median difference −22.5 pg/ml; 2^−^ HBO_2_ treatment, median difference −20.4 pg/ml). Patients presenting with shock had significantly higher baseline cytokines values compared to non‐shock patients (TNF‐α: 51.9 vs. 23.6, IL‐1β: 1.39 vs 0.61, IL‐6: 542.9 vs. 57.5, IL‐10: 21.7 vs. 3.3 and G‐CSF: 246.3 vs. 11.8 pg/ml; all *p* < 0.001). Longitudinal analyses demonstrated higher concentrations in septic shock patients and those receiving renal‐replacement therapy. All cytokines were significantly correlated to SAPS II, SOFA score, and blood lactate. In adjusted analysis, high baseline G‐CSF was associated with 30‐day mortality (OR 2.83, 95% CI: 1.01–8.00, *p* = 0.047).

**Conclusion:**

In patients with NSTI, HBO_2_ treatment may induce immunomodulatory effects by decreasing plasma G‐CSF and IL‐6. High levels of inflammatory markers were associated with disease severity, whereas high baseline G‐CSF was associated with increased 30‐day mortality.

## Graphical abstract

In necrotizing soft‐tissue infections, the inflammatory response at admission is immense with a rapid decline during the first 3 days. Hyperbaric oxygen treatment may induce immunomodulatory effects by decreasing IL‐6 and G‐CSF potentially limiting the risk of collateral damage.

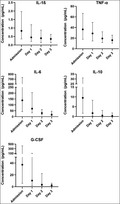

## 1 INTRODUCTION

Necrotizing soft‐tissue infection (NSTI) is a serious and life‐threatening disease. NSTI is characterized by rapidly progressing soft‐tissue inflammation and necrosis (Stevens & Bryant, 2017). NSTI is rare, with an estimated annual incidence of 4.5 cases per 100,000 in the United States (Soltani et al., 2014) and 1.99 in Denmark (Hedetoft et al., 2020). Prompt and repeated surgical debridement; antibiotic therapy and supportive intensive care are the mainstays in NSTI treatment. Despite rigorous treatment, patients with NSTI have high mortality rates, risk of amputation and often protracted rehabilitation stays (Hakkarainen et al., 2014).

Hyperbaric oxygen (HBO_2_) treatment might serve as an adjunctive to the multidisciplinary course of treatment (Anderson & Jacoby, 2019; Mathieu et al., 2017). Retrospective observational studies have shown improved survival in patients with NSTI when HBO_2_ treatment is given as an adjunct to regular treatment (Devaney et al., 2015; Shaw et al., 2014), and register‐based studies have demonstrated lower mortality in patients who received HBO_2_ treatment (Hedetoft et al., 2020; Soh et al., 2012). Although patients with NSTI might benefit from HBO_2_ treatment, no prospective controlled studies exist (Levett et al., 2015). This could be due to the rarity of the disease, ethical concerns, limited access to HBO_2_ treatment, and a lack of pharmaceutical interest in the funding of such studies using a non‐patented drug.

The antimicrobial mechanisms of action of HBO_2_ treatment is diverse and includes many specific actions. HBO_2_ treatment induces several different physiological and biochemical responses. Three main antimicrobial mechanisms have been described including direct antimicrobial effects in the result of HBO_2_‐derived formation of reactive oxygen species, synergistic effects with antibiotics, and immune‐modulatory effects (Memar et al., 2019; Thom, 2009). First, the formation of reactive oxygen species generates oxidative stress which is fundamental to HBO_2_ treatment (Thom, 2009). Oxidative stress by the formation of reactive oxygen species and reactive nitrogen species is in general believed to be destructive to bacterial DNA, RNA, proteins, and lipids (Memar et al., 2018). Second, HBO_2_ treatment induces aerobic metabolism of the bacteria while reoxygenating the O_2_‐depleted tissue (Jensen et al., 2019; Lerche et al., 2017). This factor has shown to be critical since some antibiotics (β‐lactams, aminoglycosides, and quinolones) are dependent on aerobic metabolism for an optimal effect (Memar et al., 2019). In that respect, HBO_2_ treatment has been shown to enhance the efficacy of tobramycin in *Staphylococcus aureus* endocarditis (Lerche et al., 2017) and ciprofloxacin in *Pseudomonas aeruginosa* biofilm infected tissue (Kolpen et al., 2016). Both tissue hypoxia and biofilm formation are pathologies present in NSTI patients (Korhonen et al., 2000; Siemens et al., 2016; Wang & Hung, 2004) and a target for correction by employing HBO_2_ treatment (Camporesi & Bosco, 2014; Jensen et al., 2019). Third, HBO_2_ treatment has shown to induce substantial effects on the expression of immune‐modulatory cytokines by decreasing proinflammatory cytokines such as IL‐1, IL‐6, and TNF‐α (Weisz et al., 1997) and elevating the anti‐inflammatory cytokine IL‐10 (Pan et al., 2013).

NSTI may arise either through a defined portal of entry or be cryptogenic without any breach of the skin, and either evolutions give rise to an excessive inflammatory response and promote platelet–leukocyte aggregation consequently causing endothelial damage, vascular occlusion, and widespread necrosis in the deep tissue (Stevens & Bryant, 2017). Particularly, the toxin‐induced inflammatory response may have a central role in both tissue pathology and systemic toxicity (Johansson et al., 2010; Morgan, 2010). Plasma levels of proinflammatory and anti‐inflammatory cytokines are elevated in sepsis non‐survivors (Pierrakos & Vincent, 2010). However, only a few prospective studies have investigated cytokine levels in NSTI patients (Hansen et al., 2017; Lungstras‐Bufler et al., 2004; Rodriguez et al., 2006) demonstrating IL‐1β and IL‐10 to be associated with 30‐day mortality and IL‐6 to be associated with disease severity in NSTI (Hansen et al., 2017). In this respect, it is important to note that HBO_2_ treatment has been proposed to have regulatory effects on the expression of IL‐1β during infections (Lerche et al., 2017). Likewise, in experimental sepsis in rats HBO_2_ treatment has shown to stimulate immune‐modulatory activities including IL‐10 modulation resulting in improved survival (Bærnthsen et al., 2017; Buras et al., 2006), but timing and dosage are essential factors for the overall outcome (Bærnthsen et al., 2017; Buras et al., 2006; Lerche et al., 2017).

The lack of clinical studies, the inconsistency in the use of HBO_2_ treatment and the uncertainties surrounding the mechanisms of action all highlight the need for studies describing the potential effects of HBO_2_ treatment in NSTI treatment. Of particular relevance is the question if HBO_2_ treatment can induce immune‐modulatory effects in patients with NSTI as experimental studies have suggested (Bærnthsen et al., 2017; Buras et al., 2006; Lerche et al., 2017). Therefore, we aimed to evaluate the plasma levels of inflammatory cytokines in patients with NSTI during the first 3 days after admission. We focused primarily on variations in IL‐6 before and after HBO_2_ treatment. We hypothesized that HBO_2_ treatment reduces plasma levels of IL‐6 and that high levels of IL‐6 at admission are associated with disease severity and 30‐day mortality. Secondarily, we focused on the possible HBO_2_‐mediated fluctuations in TNF‐α, IL‐1β, IL‐10, and granulocyte colony‐stimulating factor (G‐CSF) and their association with disease severity and mortality.

## 2 MATERIALS AND METHODS

### 2.1 Study design

The present study was a sub‐study of the Danish cohort from the international, prospective, observational cohort‐study (INFECT, ClinicalTrials.gov number; NCT01790698) including patients with NSTI admitted to Rigshospitalet, Copenhagen University Hospital, Denmark between February 2013 and March 2017 in which some data have been reported elsewhere (Madsen et al., 2019). We included patients aged 18 years or older after diagnosis of NSTI was confirmed by a surgeon at either the primary operation or at revision. We excluded patients in whom the surgery revealed no NSTI.

### 2.2 Patient management

Patients were treated according to a standardized multidisciplinary course including frequent surgical debridement (three revisions during first 24 h. Thereafter repeated as necessary), initial broad‐spectrum antibiotics (meropenem, ciprofloxacin, and clindamycin), supportive intensive care, immunoglobulin therapy if indicated (considered in patients with septic shock and Group A‐*Streptococcus* infection, 25 g/day for 3 days), and HBO_2_ treatment (at least three sessions of 90 min at 284 kPa, preferably two sessions within 24 h from admission and minimum three sessions within 72 h; Madsen et al., 2019).

### 2.3 Data collection

Patients had blood sampled from either an arterial or central venous line into an ethylenediaminetetraacetic acid sample tube upon admission (baseline) and at each of the following 3 days (all between 08:00 and 12:00 hours). Moreover, blood was sampled immediately before and after each session of HBO_2_ treatment. Samples were put on ice and centrifugated within 40 min. Plasma was collected and stored at −80°C until analysis. Predefined clinical data, including patients’ characteristics, Simplified Acute Physiology Score (SAPS)‐II, microbiological findings, and supportive modalities were registered into an electronic clinical database by dedicated personnel (Madsen et al., 2018).

### 2.4 Multiplex bead array assays

All samples were studied by magnetic bead suspension array using a premixed 5‐plex panel (Bio‐Rad Laboratories) according to the manufacturer's instructions except that the samples were diluted in 1:3. The premixed 5‐plex panel contained tumor necrosis factor‐α (TNF‐α), interleukin 1β (IL‐1β), interleukin 6 (IL‐6), interleukin 10 (IL‐10), and G‐CSF. Nine‐point standard curves were generated for each cytokine. All samples were analyzed using Bio‐Plex 100 System, and the concentrations were calculated using the Bio‐Plex Manager 6.0 (Bio‐Rad Laboratories). Extrapolated concentrations were used if the values fell outside the 9‐point standard curve. However, if values could not be extrapolated, values below limit were set to lowest extrapolated value on the specific panel. No values were above the detection limit. All samples from each patient were analyzed on the same panel.

### 2.5 Outcomes

The primary outcome was difference in IL‐6 before and after HBO_2_ treatment. Secondary analyses included differences in TNF‐α, IL‐1β, IL‐10, and G‐CSF before and after HBO_2_ treatment assessed in the entire cohort and in subgroups of patients with the presence of Group A‐*Streptococcus* or anaerobic species. Furthermore, the association of baseline cytokine concentration with SAPS II, SOFA score at admission, serum lactate, use of renal replacement therapy (RRT) (any use within first 7 days of ICU admission), amputation (in patients with infection of an extremity), and 30‐day all‐cause mortality were evaluated.

### 2.6 Statistics

Continuous data are presented as medians (interquartile range, IQR) and categorical data as absolute numbers (%). Testing for normality was assessed with the Shapiro–Wilks test. As data were not normally distributed, continuous data were compared using Wilcoxon rank sum test for non‐paired data and Wilcoxon signed‐rank test for paired data (before/after HBO_2_ treatment) with adjusted *P* values using Benjamini–Hochberg do to multiple comparisons. Longitudinal cytokine concentrations (admission, days 1, 2, and 3) were presented as the area under the curve (AUC) and compared using the Wilcoxon rank sum test. We assessed correlations by Spearman's rank correlation test. Receiver operating characteristic (ROC) curves were analyzed and ROC‐AUC, sensitivity, specificity, positive‐predictive value, and negative‐predictive value were reported for baseline cytokines level on 30‐day mortality. Logistic regression analyses with odds ratio (OR) and 95% confidence interval (95% CI) were performed to evaluate the association between baseline cytokine levels and 30‐day mortality and adjusted for differences in age, sex, comorbidities, and SOFA score at admission. We used the Youden Index optimal cutoff point to categorize low and high cytokine levels in logistic analyses. Patients who were lost to follow‐up were excluded from entering survival analyses. *p*‐values were reported as exact unless <0.001. *p*‐values below 0.05 were considered statistically significant. Statistical analyses were performed using R v.3.3.2 (The R Foundation for Statistical Computing Platform) with additional RStudio v.1.0.136 (Rstudio, Inc.) software attached. Figures were created using GraphPad Prism 8.0.2 (GraphPad Inc.).

### 2.7 Ethics

The study abided the principles outlined in the Declaration of Helsinki. The study was approved by the Regional Ethical Committee of Capital Region (H‐19016085) and The Danish Data Protection Agency (VD‐2019‐179). Written informed content was obtained from all patients or their legal surrogates. The Strengthening the Reporting of Observational Studies in Epidemiology (STROBE) guidelines were followed in the drafting of this manuscript (Elm et al., 2007).

## 3 RESULTS

**FIGURE 1:**
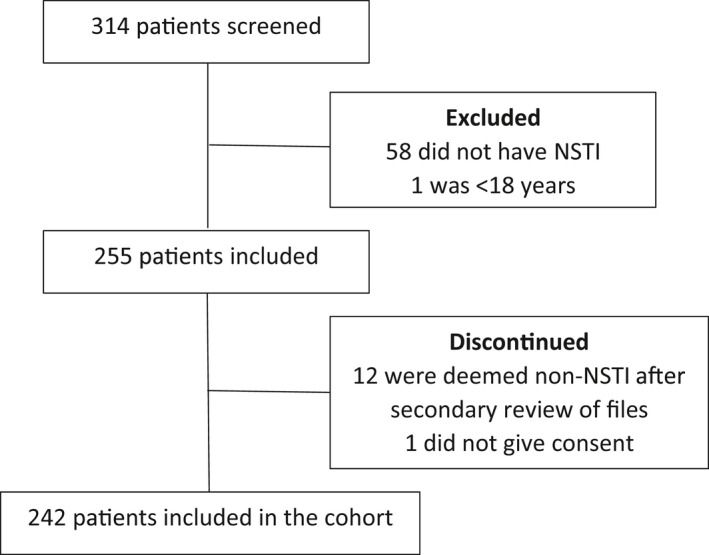
Flow chart of patients included in the study. Patients with suspected NSTI were screened for eligibility. Patients were excluded if they did not meet the criteria of inclusion. After inclusion, patients’ files were reviewed and 12 were deemed non‐NSTI due to no intraoperative signs of necrotizing soft tissue infection. One patient was discontinued as informed consent was not obtainable

**FIGURE 2:**
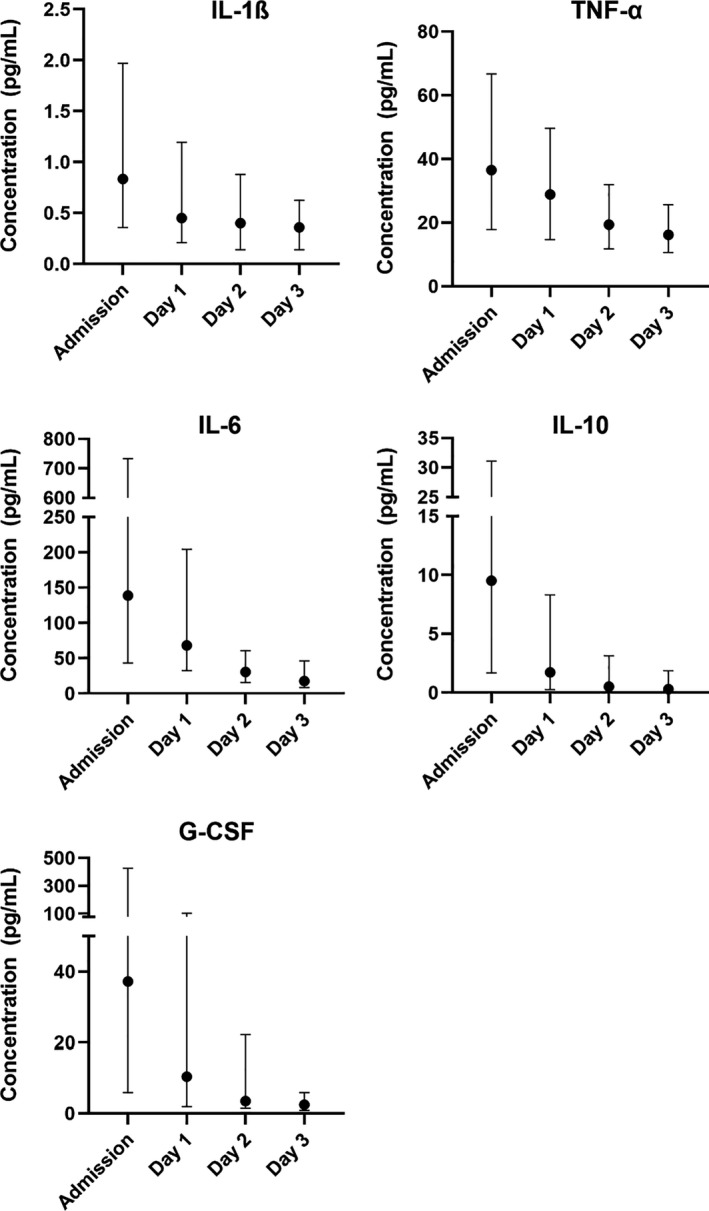
Cytokine concentrations at admission (*n* = 242), day 1, day 2 and day 3. Data are plotted as medians with interquartile range. Note the two‐segmented *y*‐axis for IL‐6, IL‐10 and G‐CSF

We included 242 patients with confirmed NSTI between February 2013 and March 2017 (Figure 1). Patients’ characteristics, including baseline cytokine levels, laboratory values, microbial findings, clinical severity scores, and outcomes, are presented in Table 1. In total, 209 (86%) patients received at least one session of HBO_2_ treatment with a median number of three sessions (3–3). Three patients were lost to follow‐up at day 30 (98.8% follow‐up). All cytokines demonstrated highest levels at admission with a moderate decrease toward days 1, 2, and 3 (Figure 2).

**TABLE 1:** Patients characteristics

	NSTI (*n* = 242)
Age (years)	62 (51–61)
Sex, male	144 (60)
BMI (kg/m^2^)	26 (24–31)
Comorbidities	
Cardiovascular disease	110 (45)
Chronic kidney disease	17 (7)
COPD	30 (12)
Diabetes (type I and II)	68 (28)
Immune deficiency	12 (5)
Chronic liver disease	14 (6)
Malignancy	19 (8)
Peripheral vascular disease	31 (13)
Rheumatoid disease	16 (7)
No comorbidities	68 (28)
Microbiological findings	
Monomicrobial infections (*n* = 96, 40%)	
Group A‐*Streptococcus*	50 (52)
Group B‐*Streptococcus*	1 (1)
Group C/G‐*Streptococcus*	9 (9)
*Staphylococcus aureus*	11 (11)
Aerobic gram‐negative species	15 (16)
Clostridium species	5 (5)
Anaerobic bacteria	3 (3)
Other streptococci	2 (2)
Polymicrobial infections (*n* = 126, 52%)	
With presence of Group A‐*Streptococcus*	8 (6)
With presence of Group B‐*Streptococcus*	10 (8)
With presence of Group C/G‐*Streptococcus*	8 (6)
With presence of *S*. *aureus*	9 (7)
With presence of *Clostridium* sp.	2 (2)
Other polymicrobial infections	89 (71)
Negative findings	20 (8)
Cytokines (pg/ml)	
IL‐6	138.6 (43.3–733.1)
IL‐1β	0.8 (0.4–2.0)
TNF‐α	36.5 (17.8–66.7)
IL‐10	9.5 (1.7–31.1)
G‐CSF	37.2 (5.9–426.0)
Biochemistry	
Leukocyte count (10^9^/L)	16.6 (11.1–23.4)
C‐reactive protein (mg/L)	226 (154–309)
Creatinine (µmol/L)	109 (74–192)
Lactate (mmol/L)	2.2 (1.3–3.9)
Other	
SOFA score^a^	8 (6–10)
SAPS II^b^	44 (35–55)
Septic shock upon admission^c^	114 (47)
Mechanical ventilation	230 (95)
Amputation within 7 days^d^	33 (14)
RRT within 7 days	42 (17)
HBOT, at any time	209 (86)
HBOT, number of sessions	3 (3–3)
30‐day mortality, % (95% CI)	17 (13–23)
90‐day mortality, % (95% CI)^e^	23 (17–28)

Continuous data are presented as medians (interquartile range, IQR) and categorical data as absolute numbers (%). Blood samples were obtained at arrival to specialized hospital.

Abbreviations: COPD, Chronic obstructive pulmonary disease; HBOT, Hyperbaric oxygen therapy; RRT. Renal‐replacement therapy.

^a^ Sequential Organ Failure Assessment (SOFA) score (Day 1); data were missing for 9 (4%) patients.

^b^ Simplified Acute Physiology Score II (SAPS II); data were missing for 9 (4%) patients.

^c^ Septic shock is defined as lactate >2 mmol/L and use of vasopressor or inotrope; data were missing for 1 patient (>0.01%).

^d^ One hundred twelve patients with infection located to the extremities.

^e^ Three patients were lost to follow‐up at day 90.

### 3.1 HBO_2_ treatment and cytokine response

In paired‐analyses, a statistically significant increase in IL‐6 was observed after the first HBO_2_ treatment compared to samples taken before the first HBO_2_ treatment (median 7.5 pg/ml, 95% CI 2.4–15.1, *p* = 0.008), and after the second HBO_2_ treatment a decrease in IL‐6 was observed compared to samples taken before the second HBO_2_ treatment (median −3.2 pg/ml, 95% CI −5.9 to −1.1, *p* = 0.01). No differences were found in IL‐6 before/after the third HBO_2_ treatment. G‐CSF showed a consistent decrease before and after all HBO_2_ treatments (Table 2). No difference in plasma levels was observed for the remaining cytokines (Table 2).

**TABLE 2:** Plasma cytokine concentrations before and after first, second_,_ and third HBOT

		First HBO_2_				Second HBO_2_				Third HBO^2^			
	pg/ml	Before	After	MD	*p* (Adj.)	Before	After	MD	*p* (Adj.)	Before	After	MD	*p* (Adj.)
All patients (n = 209)	IL‐1β	0.66 (0.23–1.23)	0.68 (0.24–1.45)	−0.05 (−0.19 to 0.07)	.55	0.37 (0.14–0.91)	0.39 (0.14–0.93)	0.01 (−0.11 to 0.14)	.78	0.39 (0.17–0.85)	0.41 (0.19–1.01)	0.05 (−0.08 to 0.19)	.53
	TNF‐α	31.8 (18.2–55.8)	30.4 (18.4–50.2)	−1.0 (−2.6 to 0.5)	.46	22.3 (14.8–40.5)	23.1 (13.9–40.5)	−0.2 (−1.4 to 1.0)	.77	19.2 (10.8–30.9)	17.5 (12.6–30.0)	0.5 (−0.5 to 1.7)	.47
	IL‐6	84.4 (37.1–237.6)	87.1 (39.2–216.7)	7.5 (2.4–15.1)	.008	47.1 (25.2–113.3)	45.0 (22.0–119.1)	−3.2 (−5.9 to −1.1)	.01	27.8 (13.4–53.2)	25.4 (13.1–53.3)	−0.8 (−2.0 to 0.5)	.46
	IL‐10	3.7 (0.3–11.7)	3.0 (0.5–11.4)	−0.8 (−1.7 to −0.2)	.12	0.8 (0.3–4.3)	1.2 (0.3–4.5)	0.2 (−0.4 to 0.8)	.68	0.3 (0.2–2.7)	0.3 (0.2–2.4)	0.3 (−0.3 to 1.1)	.47
	G‐CSF	21.2 (2.9–179.8)	19.2 (3.0–118.9)	−22.5 (−46.6 to −10.5)	<.001	5.9 (1.3–51.8)	5.7 (1.3–36.3)	−20.4 (−37.5 to −8.6)	<.001	2.7 (0.9–6.2)	2.7 (1.0–6.7)	−2.6 (−10.1 to 1.7)	.47
Streptococcus Group A (*n* = 51)	IL‐1β	0.81 (0.40–1.69)	0.78 (0.19–1.79)	−0.36 (−0.70 to −0.05)	.049	0.31 (0.12–0.80)	0.55 (0.16–0.83)	0.06 (−0.23 to 0.39)	.67	0.28 (0.12–0.77)	0.40 (0.12–0.83)	0.08 (−0.16 to 0.35)	.51
	TNF‐α	39.3 (26.2–64.1)	36.6 (24.6–64.5)	−1.3 (−4.3 to 1.5)	.48	29.7 (17.4–50.7)	29.0 (17.3–51.7)	1.5 (−2.5 to 4.7)	.49	19.7 (14.7–34.1)	23.7 (14.8–35.7)	1.6 (−2.0 to 5.8)	.50
	IL‐6	184.3 (60.6–595.7)	172.6 (45.2–454.9)	−29.5 (−79.9 to −8.0)	.01	49.7 (25.2–162.0)	45.7 (22.1–136.5)	−7.6 (−26.0 to −2.4)	.01	19.9 (12.1–35.9)	18.2 (10.8–21.3)	−2.1 (−4.2 to −0.5)	.03
	IL‐10	5.5 (1.5–17.8)	5.8 (1.3–18.6)	−1.0 (−3.3 to 0.3)	.22	1.3 (0.3–4.6)	2.3 (0.7–4.7)	0.3 (−1.5 to 1.7)	.79	0.3 (0.2–1.6)	1.3 (0.3–2.6)	1.4 (−0.3 to 3.3)	.18
	G‐CSF	261.7 (38.7–1778.5)	127.3 (26.3–1771.0)	−106.9 (−346.7 to −35.6)	.003	79.0 (12.8–185.9)	45.3 (8.7–159.6)	−51.4 (−101.1 to −25.6)	.002	4.7 (2.0–62.0)	4.7 (1.8–33.4)	−10.9 (−25.8 to −1.9)	.04
Anaerobic (*n* = 78)	IL‐1β	0.68 (0.23–1.31)	0.78 (0.32–1.60)	0.03 (−0.17 to 0.25)	.68	0.55 (0.21–1.18)	0.40 (0.21–0.97)	−0.12 (−0.33 to 0.06)	.61	0.51 (0.16–1.08)	0.44 (0.22–1.16)	0.10 (−0.2 to 0.38)	.58
	TNF‐α	34.3 (20.6–54.4)	29.3 (18.5–55.8)	−3.0 (−6.0 to −0.4)	.13	21.03 (15.7–37.4)	23.8 (15.0–39.3)	−0.67 (−2.8 to 1.4)	.78	17.9 (9.5–30.7)	17.5 (10.4–27.8)	0.91 (−0.8 to 4.8)	.46
	IL‐6	84.0 (37.1–202.4)	75.2 (35.6–174.4)	−6.8 (−18.7 to 0.1)	.18	43.0 (26.1–81.6)	49.5 (23.3–73.6)	−2.0 (−5.3 to 1.0)	.39	26.7 (13.4–40.1)	23.7 (15.7–44.1)	1.0 (−1.2 to 3.9)	.56
	IL‐10	3.9 (0.3–18.5)	3.2 (0.3–13.5)	−1.8 (−4.6 to −0.3)	.13	0.52 (0.2–3.7)	0.9 (0.3–2.5)	0.57 (−0.3 to 1.6)	.39	0.3 (0.2–2.4)	0.3 (0.2–2.4)	0.4 (−0.7 to 2.9)	.61
	G‐CSF	22.6 (2.5–79.3)	16.3 (3.1–69.5)	−10.6 (−24.5 to 0.4)	.18	4.9 (1.2–24.5)	4.3 (1.3–21.1)	−4.7 (−14.1 to 1.3)	.25	2.5 (0.9–4.9)	2.5 (1.0–5.9)	6.9 (0.2–20.2)	.12

Concentrations shown in pg/ml. Values presented for all HBO_2_‐treated patients (1 treatment: *n* = 209; 2 treatments: *n* = 190 and 3 treatments: *n* = 164 patients) sub‐groups of patients with presence of either Group A‐*Streptococcus* (1 treatment; *n* = 51. 2 treatments; *n* = 45 and 3 treatments; *n* = 36) or anaerobic species (1 treatment; *n* = 78. 2 treatments; *n* = 70 and 3 treatments; *n* = 61), in tissue and/or blood. Data are presented as group medians and interquartile range. Paired analyses (before/after HBO_2_) with median differences (95% CI) evaluated by Wilcoxon Signed‐Rank Test with Benjamini–Hochberg adjusted *p*‐values due to multiple comparisons.

Abbreviation: MD, Median difference.

Subgroup analyses of patients with *Group A*‐*Streptococcus* revealed a significant and consistent decrease in IL‐6 both after the first, second, and third HBO_2_ treatment compared to the level before the, respectively, treatment (Table 2). Furthermore, significant differences in IL‐1β after the first HBO_2_ treatment and G‐CSF at both first, second, and third HBO_2_ treatment were observed (Table 2). In analyses of patients with anaerobic species, no significant changes were observed (Table 2).

### 3.2 Cytokines and NSTI severity

Patients with NSTI and septic shock at admission (*n* = 114, 47%) had significantly higher baseline cytokine levels compared to non‐shock patients (Table 3). Likewise, patients receiving RRT within the first 7 days from admission had significantly higher baseline values compared to non‐RRT patients (Table 3). However, no significant differences were found between patients receiving amputation compared to non‐amputated NSTI patients (Table 3).

**TABLE 3:** Cytokine concentrations at admission (top) and longitudinal concentrations (AUC) during first 3 days from admission (bottom)

	IL‐1β		TNF‐α		IL‐6		IL‐10		G‐CSF	
Admission										
Shock	1.39 (0.64–3.89)	*p* < .001	51.9 (31.6–89.1)	*p* < .001	542.9 (143.0–3,809.1)	*p* < .001	21.7 (9.7–63.7)	*p* < .001	246.3 (33.2–5,171.0)	*p* < .001
Non‐shock	0.61 (0.22–1.35)		23.6 (14.2–47.9)		57.5 (30.0–169.6)		3.3 (0.3–10.0)		11.8 (3.3–51.1)	
RRT	1.57 (0.76–3.68)	*p* = .009	64.1 (39.8–137.0)	*p* < .001	1145.0 (193.1–7,495.2)	*p* < .001	28.0 (9.7–113.3)	*p* = .002	709.4 (37.4–5,456.1)	*p* < .001
Non‐RRT	0.82 (0.31–1.90)		36.5 (16.6–69.6)		140.5 (54.5–462.6)		9.5 (2.3–26.9)		34.7 (3.7–245.7)	
Amputated	0.83 (0.26–1.9)	*p* = .99	39.9 (16.7–97.4)	*p* = .49	353.6 (49.3–2,361.9)	*p* = .09	21.5 (5.6–36.7)	*p* = .12	102.8 (7.3–5,641.4)	*p* = .25
Non‐amputated	0.84 (0.36–2.0)		36.3 (18.1–60.8)		113.4 (42.8–517.2)		8.6 (1.4–24.7)		34.6 (5.5–271.9)	
Longitudinal AUC										
Shock	2.04 (0.97–3.72)	*p* = .007	95.5 (51.5–160.4)	*p* < .001	543.9 (131.2–2,794.2	*p* < .001	18.3 (8.0–42.2)	*p* < .001	175.5 (24.1–4,220.2)	*p* < .001
Non‐shock	1.33 (0.75–2.47		53.7 (34.6–89.2)		138.7 (67.7–291.2)		4.1 (1.4–12.7)		15.5 (7.3–58.0)	
RRT	2.80 (1.47–5.79)	*p* = .007	160.9 (107.0–210.4)	*p* < .001	1214.5 (280.9–6,884.5)	*p* = .001	40.1 (12.0–146.1)	*p* < .001	780.5 (64.2–13,254.5)	*p* < .001
Non‐RRT	1.71 (0.72–3.19)		71.0 (43.2–272.7)		247.8 (130.9–545.6)		10.3 (3.0–28.5)		34.0 (8.8–234.5)	
Amputated	2.04 (0.66–3.19)	*p* = 0.56	83.0 (36.3–159.3)	*p* = .45	364.0 (117.3–2016.0)	*p* = .08	19.2 (3.9–41.1)	*p = *.10	87.8 (8.1–3,471.0)	*p* = .27
Non‐amputated	1.59 (0.77–3.03)		68.6 (38.9–114.1)		206.6 (80.1–644.3)		8.0 (1.8–24.8)		33.6 (8.9–244.4)s	

Concentrations in pg/ml. Results presented according to patients presenting with shock at admission (114 shock vs 128 non‐shock), receiving RRT within first 7 days (42 RRT vs. 200 non‐RRT) and receiving amputations within first 7 days (33 amputated vs. 79 non‐amputated; only patients with NSTI located to the extremities (*n* = 112) were included). Septic shock defined as lactate >2 mmol/L and use of vasopressor or inotrope. Data are presented as medians (interquartile range, IQR). Statistical comparisons evaluated by Wilcoxon Rank Sum Test.

Abbreviations: AUC, area under curve; RRT, renal‐replacement therapy.

All baseline cytokines concentration showed a significant correlation with lactate, SAPS II, and SOFA score (Table 4).

**TABLE 4:** Spearman rank correlation between severity of disease and baseline biomarker level

	SAPS II		SOFA		Lactate	
	Rho	*p*	Rho	*p*	Rho	*p*
IL‐1β	0.23	.001	0.25	<.001	0.41	<.001
TNF‐α	0.38	<.001	0.46	<.001	0.50	<.001
IL‐6	0.32	<.001	0.52	<.001	0.64	<.001
IL‐10	0.32	<.001	0.46	<.001	0.55	<.001
G‐CSF	0.24	.001	0.40	<.001	0.54	<.001

Abbreviations: SAPS II, Simplified Acute Physiology Score II; SOFA, Sequential Organ Failure Assessment.

Examining longitudinal concentrations by AUC; IL‐1β, TNF‐α, IL‐6, IL‐10, and G‐CSF showed a median AUC of 1.72 (0.77–3.10), 69.0 (38.8–124.3), 224.8 (81.9–786.2), 9.5 (2.0–29.1), and 35.5 (8.7–310.3), respectively. In longitudinal analyses, patients presenting with shock at admission had significantly higher AUC in all cytokines compared to non‐shock patients (Table 3). This was also found in patients receiving RRT compared to non‐RRT patients (Table 3). No differences were found in longitudinal AUC between patients receiving amputation and non‐amputated patients (Table 3).

### 3.3 Cytokines and 30‐day mortality

**FIGURE 3:**
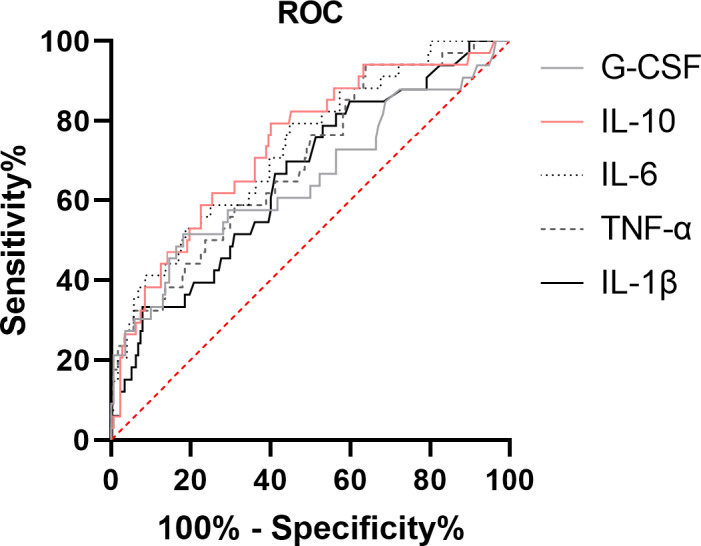
Receiver operating characteristic curve of 30‐day mortality in patients with necrotizing soft‐tissue infections for the pro‐ and anti‐inflammatory cytokines

All cytokines showed good moderate ROC‐AUC (Figure 3) with IL‐10 demonstrating the highest AUC of 0.74 (95% CI 0.64–0.84) (Table 5). The optimal cut‐off point (Youden Index) for IL‐1β, TNF‐α, IL‐6, IL‐10, and G‐CSF were found to be 0.86, 20.86, 133.5, 10.1, and 446.9, respectively. In unadjusted logistic regression models, both IL‐1β, TNF‐α, IL‐6, IL‐10, and G‐CSF showed to be associated with 30‐day mortality (Table 6). These results were unaltered when adjusting for age, sex, and comorbidity, however, only G‐CSF was statistically significantly associated with 30‐day mortality when additionally adjusted for SOFA score (Table 6). Accuracy for prediction of 30‐day mortality according to high versus low baseline cytokine levels are presented in Table 5.

**TABLE 5:** Accuracy of high baseline biomarker (defined by being above the optimal cut‐off point) level in predicting 30‐day mortality

	IL‐1β	TNF‐α	IL‐6	IL‐10	G‐CSF
Sensitivity	0.71 (0.51–0.85)	0.94 (0.80–0.99)	0.79 (0.62–0.91)	0.79 (0.62–0.91)	0.52 (0.34–0.69)
Specificity	0.56 (0.45–0.63)	0.34 (0.27–0.42)	0.55 (0.47–0.62)	0.60 (0.52–0.67)	0.81 (0.74–0.87)
PPV	0.24 (0.16–0.33)	0.22 (0.15–0.29)	0.25 (0.17–0.35)	0.28 (0.19–0.37)	0.35 (0.22–0.50)
NPV	0.91 (0.84–0.96)	0.97 (0.89–1.00)	0.93 (0.86–0.97)	0.94 (0.88–0.97)	0.90 (0.84–0.94)
AUC‐ROC	0.67 (0.56–0.77)	0.70 (0.59–0.80)	0.73 (0.63–0.83)	0.74 (0.64–0.84)	0.65 (0.55–0.76)

Data are presented as fractions (95% confidence interval).

Abbreviations: AUC‐ROC, area under the receiver operating characteristics curve; NPV, negative predictive value; PPV, positive predictive value.

**TABLE 6:** Univariate and multivariate logistic regression analyses of 30‐day mortality based on low versus high baseline cytokine levels according to the optimal cut‐off values

pg/ml	Unadjusted			Adjusted analysis: age, sex and comorbidities			Adjusted analysis: age, sex, comorbidities and SOFA score		
	OR	95% CI	*P*	OR	95% CI	*p*	OR	95% CI	*p*
IL‐1β									
Low ≤0.86	1 Ref.			1 Ref.			1 Ref.		
High >0.86	3.05	1.41–7.03	.006	3.54	1.56–8.64	.004	2.60	0.99–7.35	.059
TNF‐α									
Low ≤20.86		1 Ref.			1 Ref.			1 Ref.	
High >20.86	8.41	2.44–53.01	.004	7.20	2.03–45.97	.009	3.42	0.77–2.52	.15
IL‐6									
Low ≤133.5		1 Ref.			1 Ref.			1 Ref.	
High >133.5	4.63	2.01–12.05	<.001	4.42	1.87–11.82	.001	1.32	0.45–4.05	.61
IL‐10									
Low ≤10.1		1 Ref.			1 Ref.			1 Ref.	
High >10.1	5.76	2.50–15.01	<.001	5.80	2.43–15.63	<.001	2.39	0.85–7.22	.11
G‐CSF									
Low ≤446.9		1 Ref.			1 Ref.			1 Ref.	
High >446.9	4.58	2.09–10.13	<.001	6.16	2.58–15.32	<.001	2.83	1.01–8.00	.047

Abbreviations: CI, confidence interval; OR, odds ratio; SOFA, sequential organ failure assessment.

Longitudinal AUC analyses showed significant differences between survivors and non‐survivors according to IL‐6 (206.6 [74.7–544.8] vs. 547.6 [114.0–4,370.3], *p* = 0.007) and IL‐10 (7.09 [1.68–23.09] vs. 20.96 [5.32–142.20], *p* = 0.001). However, no differences were found according to IL‐1β (1.65 [0.76–2.87] vs. 1.81 [0.78–3.41], *p* = 0.63), TNF‐α (68.8 [40.7–120.3] vs. 71.9 [29.9–138.9], *p* = 0.95) or G‐CSF (35.5 [8.9–233.6] vs. 87.8 [8.7–9,853.7], *p* = 0.17).

## 4 DISCUSSION

In this study evaluating several pro‐ and anti‐inflammatory cytokines in patients with NSTI, IL‐6 levels were decreased after adjunctive HBO_2_ treatment in patients with *Group A*‐*Streptococcus*, and decreased G‐CSF levels in general. Moreover, we found high levels of IL‐1β, TNF‐α, IL‐6, IL‐10, and G‐CSF to be associated with severity of disease as represented by higher cytokine levels among patients with septic shock and positive correlations with SAPS II, SOFA score, and lactate. High baseline G‐CSF demonstrated to be an independent risk factor of 30‐day mortality.

Cytokine assessment may be a valuable tool for the treating clinician and may be useful for prognosis prediction and clinical decision‐making (Bozza et al., 2005; Pierrakos et al., 2020). In patients with severe sepsis and septic shock; IL‐1β, IL‐6, and G‐CSF have demonstrated good accuracy for predicting early mortality while IL‐6 and G‐CSF showed to be predictive of worsening of organ dysfunction (Bozza et al., 2007). In addition, both IL‐1β, IL‐6, and G‐CSF have shown to be elevated in septic non‐survivors compared to survivors during the first 3 days upon admission (Mera et al., 2011). Interestingly, cytokine network analyses have revealed a network formed by IL‐1β, IL‐6, and IL‐10 during the first day of sepsis, suggesting that these cytokines may take a crucial role in the acute phase of sepsis (Matsumoto et al., 2018). The present results demonstrated that G‐CSF was significantly associated with 30‐day mortality in patients with NSTI and is in accordance with the present literature in septic patients. However, of ample notice, G‐CSF and IL‐1β ROC‐AUC values in predicting 30‐day mortality did not reach an acceptable level of above 0.7, indicating that the accuracy of prediction should be cautiously interpreted (Mandrekar, 2010). Of interests, IL‐1β has previously demonstrated to be a predictor of 30‐day mortality in a smaller part of the present cohort (*n* = 159; Hansen et al., 2017), but in the present study including the entire Danish cohort (*n* = 242); IL‐1β did not reach a statistically significant level in predicting 30‐day mortality.

Cytokines have been profoundly studied in septic patients (Pierrakos et al., 2020). However, only a few studies exist on cytokine profiles in patients with NSTI indicating that particular IL‐1β, IL‐6, IL‐10, and IL‐18 may take a crucial part in NSTI pathophysiology (Hansen et al., 2017; Lungstras‐Bufler et al., 2004; Rodriguez et al., 2006). In sepsis, an excessive release of proinflammatory cytokines may cause collateral damage to the endothelium layer and progress into systemic inflammatory response syndrome, multiple organ failure with a substantially high risk of morbidity and mortality (Levi & Poll, 2017; Levi & Van Der Poll, 2013). In that context, the rapid and severe cause of disease among patients with NSTI may reflect a disproportional and excessive proinflammatory response caused by toxin production and cytokine activation (Bonne & Kadri, 2017; Johansson et al., 2010; Norrby‐Teglund et al., 2001). However, the concept of an initial uncontrolled proinflammatory response followed by an anti‐inflammatory phase seems to be a simplified model, and increasing evidence indicates that the host response is dependent on numerous complex, dynamic, and concomitant pathogen and host‐immune defense mechanisms such as the bacterial virulence and microbial load (Schulte et al., 2013; Van Der & Opal, 2008).

Sepsis‐mediated endothelial damage increases IL‐6 proliferation that causes soluble IL‐6 to bind to its receptor (IL‐6R), forming the IL‐6/IL‐6R complex (Tanaka et al., 2016). The IL‐6/IL‐6R complex activates glycoprotein 130 (Jones et al., 2011)—a signal‐transducing component placed on various tissues and cells including the endothelium—consequently triggering various of downstream effects including the production of acute‐phase proteins (Heinrich et al., 1990), inducement of complement C3 and C5a receptor (Tanaka et al., 2016), initiation of coagulation through tissue factor activation (Neumann et al., 1997) and activates the endothelium to produce IL‐6 and enhance intercellular adhesion molecule‐1 (ICAM‐1) expression resulting in increased leukocyte recruitment (Romano et al., 1997; Tanaka et al., 2016). IL‐1β, a powerful proinflammatory cytokine primarily created by activated macrophages and monocytes, acts on a variety of immune cells including the endothelium, amplifying the inflammatory process by release of proinflammatory cytokines—such as IL‐6—and reactive oxygen and nitrogen species (Schulte et al., 2013). In this context, it may be of importance to note, that both ICAM‐1 and IL‐6 are modified by HBO_2_ treatment in experimental sepsis (Bærnthsen et al., 2017; Buras et al., ,^2000^, ^2006^; Halbach et al., 2019). Some evidence indicates that G‐CSF—a hematopoietic‐cell growth factor—is involved in sepsis pathogenesis and has crucial functions on mature myeloid cells and the innate immune system during inflammation (Hamilton, 2008; Roberts & Roberts, 2005). G‐CSF is markedly increased during severe infections (Roberts & Roberts, 2005), and a linkage between colony‐stimulating factors and expression of IL‐1β and TNF‐α has earlier been suggested. The present result demonstrating high baseline G‐CSF as an independent risk factor of 30‐day mortality, suggests G‐CSF may help guide the treating physician in risk stratification as high G‐CSF at admission indicates an increased risk of dying within day 30.

It has become well‐accepted that sepsis is not a linear process (Bozza et al., 2005). Consequently, studies only assessing cytokines at admission may lack important information on pathophysiological understanding and its impact on clinical progression and outcomes. Therefore, in an effort of evaluating the overall inflammatory status, we addressed the cytokine level from admission throughout the following 3 days as AUC. To our knowledge, no studies have—to date—examined the kinetics of cytokines in NSTI, and only a few studies with septic patients have evaluated the progress of cytokines during the first days from admission (Matsumoto et al., 2018; Mera et al., 2011). In general, these studies agree with the present results demonstrating markedly highest cytokine concentrations at admission with a substantial decrease the following days. Yet, an inverse development has been observed in sepsis non‐survivors according to IL‐1β and G‐CSF demonstrating gradually increased values during the first 3 days from admission compared to sepsis survivors (Mera et al., 2011). However, in patients with NSTI we were unable to detect any differences between NSTI survivors and non‐survivors according to longitudinal AUC analyses of IL‐1β and G‐CSF.

In a large multicenter observational study including 583 patients with severe sepsis, it was demonstrated that patients with high levels of IL‐6 and IL‐10 had markedly increased risk of dying (hazard ratio of 20.52) (Kellum et al., 2007). Interestingly, these findings seem in accordance with the present longitudinal results of patients with NSTI; indicating that mortality is highest when both pro‐ and anti‐inflammatory cytokines are high.

HBO_2_ treatment induces several immunomodulatory effects that may well explain the observed cytokine fluctuations before and after HBO_2_ treatment. Studies of experimental sepsis have indicated that HBO_2_ treatment exerts its antimicrobial effect by enhancement of IL‐10, which lowers IL‐6 and thereby reduces the overall mortality in HBO_2_‐treated animals (Bærnthsen et al., 2017; Buras et al., 2006). However, we were unable to detect any fluctuations of IL‐10 after HBO_2_ treatment, but this discrepancy may well be explained by our time of blood sampling immediately after treatment which does not respect the less rapid kinetics of IL‐10 (Khatri & Caligiuri, 1998). Of notice, patients with *Group A*‐*Streptococcus* NSTI have higher rates of septic shock (Madsen et al., 2019), and HBO_2_ treatment has shown its greatest effect in the most severely ill NSTI patients (Devaney et al., 2015; Shaw et al., 2014); therefore, the present observed attenuation of IL‐6 subsequently after HBO_2_ treatment in patients with *Group A*‐*Streptococcus* may well reflect this clinical effect with a more pronounced immunomodulatory effect in the most severely ill patients. Furthermore, in patients with Crohn's disease HBO_2_ treatment has shown to modulate the proinflammatory response by an initial elevation of IL‐1 but during courses of HBO_2_ decreasing IL‐1; indicating that prolonged HBO_2_ treatment may initially exhaust monocytes of stored IL‐1 subsequently leading to an inhibition of cytokine production and/or secretion (Weisz et al., 1997). Interestingly, this HBO_2_‐mediated immunosuppressive effect does not seem to reduce the phagocytic activity of the macrophages and their bacterial defense mechanisms remain intact (Inamoto et al., 1991). G‐CSF has sparsely been investigated in relation to HBO_2_ treatment, but in patients with carbon monoxide poisoning courses of HBO_2_ treatment has shown to initial increase G‐CSF subsequent decreasing G‐CSF afterward (Schnittger et al., 2004). We found a significant reduction in G‐CSF immediately after first and second HBO_2_ treatment compared to samples before treatment which may be central in the suggested HBO_2_‐mediated immunomodulatory effect by G‐CSF’s inhibition on IL‐1β expression and the innate immune response. In that context, we demonstrated a reduction of IL‐1β after first HBO_2_ treatment in patients with *Group A*‐*Streptococcus*.

Of great notice, the present study does not include a matched control group of patients not receiving HBO_2_ treatment, thus we cannot definitely ascertain that the observed fluctuations are due to the treatment itself or could represent a general improvement in the clinical condition. However, the relatively short time period between HBO_2_ treatment and blood sampling increases the confidence that the observed effects are mediated by the treatment and not the natural cause of declination caused by gradual improvement of the patient's condition.

A series of strengths to the present study exists. First, the substantial‐high follow‐up rate of >98% increases the validity of the present results. Second, the broad inclusion and limited exclusion criteria strengthen the study's generalizability. Third, we measured cytokine concentrations in a longitudinal design thus increasing the information of the overall inflammatory response over time. Last, we analyzed a limited number of cytokines and thereby reduced the risk of chance findings. Limitations of the study include the absence of a matched control group of patients not receiving HBO_2_ treatment, and that the laboratory analysts were not blinded for patient outcome.

In conclusion, our study suggests an immune‐modulating effect of HBO_2_ treatment in NSTI patients as indicated by decreasing IL‐6 in patients with *Group A*‐*Streptococcus* and decreasing G‐CSF in general. Secondly, that high baseline levels of proinflammatory and anti‐inflammatory cytokines are associated with severity of disease and high G‐CSF at admission is associated with 30‐day mortality. Future research on the possible derived effects on the immune response during HBO_2_ treatments in general and in NSTI patients specifically are much warranted.

## CONFLICT OF INTEREST

No conflicts of interests, financial, or otherwise declared by the authors.

## AUTHOR CONTRIBUTIONS

Study planning (MH, PG, OH), laboratory analysis (MH and Biotechnician Bettina Eide Holm), data analyses (MH, OH), results interpretation (MH, PG, MM, OH), manuscript drafting (MH, OH), revision and approval of the final version of the manuscript (MH, PG, MM, OH).
